# Evolutionary dynamics of recurrent hepatocellular carcinoma under divergent immune selection pressures

**DOI:** 10.3389/fonc.2025.1537087

**Published:** 2025-08-04

**Authors:** Ankur Chakravarthy, Elisa Pasini, Xun Zhao, Jeffrey To, Shu Yi (Roxana) Shen, Sandra Fischer, Anand Ghanekar, Arndt Vogel, Robert C. Grant, Jennifer Knox, Gonzalo Sapisochin, Gregory J. Gores, Daniel De Carvalho, Mamatha Bhat

**Affiliations:** ^1^ Princess Margaret Cancer Centre, University Health Network, Toronto, ON, Canada; ^2^ Ajmera Transplant Centre, University Health Network, Toronto, ON, Canada; ^3^ Department of Pathology, University Health Network, Toronto, ON, Canada; ^4^ Division of Multi-Organ Transplant and Hepato-Pancreato-Biliary (HPB) Surgical Oncology, Department of General Surgery, University Health Network, Toronto, ON, Canada; ^5^ Division of Gastroenterology & Hepatology, Mayo Clinic, Rochester, MN, United States; ^6^ Department of Medical Biophysics, University of Toronto, Toronto, ON, Canada; ^7^ Division of Gastroenterology and Hepatology, University of Toronto, Toronto, ON, Canada; ^8^ Ajmera Transplant Centre, Toronto General Hospital Research Institute, Toronto, ON, Canada; ^9^ Institute of Medical Science, University of Toronto, Toronto, ON, Canada

**Keywords:** hepatocellular carcinoma, tumour evolution, immune selection, immune evasion, whole-exome sequencing

## Abstract

Hepatocellular carcinoma (HCC) is a highly lethal, aggressive malignancy. Little is known about the evolutionary trajectories of HCC and how clinical decision-making could be informed based on biopsies of the initial tumour. Here, we report the whole-exome sequencing of a unique series of resected HCC tumours and matched recurrences. This cohort included patients who received a liver transplant and who were immunosuppressed at time of recurrence, in comparison to patients who underwent liver resection for HCC and immunocompetent at time of recurrence, therefore facilitating analyses of immune selection in driving evolutionary divergence. We find extensive evolutionary divergence between baseline and recurrent tumours, with the majority of mutations in our cohort being private, in the process informing sampling guidelines for precision oncology in this disease. We also find no evidence that immunosuppression relaxes immune selection pressures, given the absence of a genomic footprint reflecting the presentation of neoantigens or altered dynamics of genomic evolution. We attribute this to the presence of genetic lesions that confer the capabilities of immune evasion in these tumours prior to transplantation, and then validate the link between immune selection pressures and the emergence of these lesions in publicly available HCC datasets. Our findings point to HCC as a cancer with extensive evolutionary divergence over time, partly defined by an irreversible, genetically determined trajectory towards immune escape.

## Introduction

Hepatocellular carcinoma (HCC) is a high-fatality cancer represents and approximately 70% of all primary liver cancers and ranks as the sixth most frequently diagnosed cancer globally (1). In the background of chronic liver disease of various aetiologies, the ongoing cycle of injury and compensatory liver regeneration results in accumulation of cancer-causing mutations. HCC treatment is challenging as clinicians must take into consideration not only the characteristics of the primary tumour, but also the patient’s functional status and underlying liver disease that may limit therapeutic options (2, 3). Therapies such as resection or systemic therapy that would otherwise be feasible in other cancers cannot be tolerated by many patients. Carefully selected patients with HCC may be candidates for liver transplantation as a unique paradigm in solid organ transplant medicine, wherein transplantation is offered as curative treatment (4, 5). Despite careful patient selection, recurrence of the primary tumour occurs in 20% and is often aggressive with a median survival of 12 months (6, 7). Clinical predictors of recurrence include tumour burden on the explant (excised, diseased liver), presence of vascular invasion, and elevated alpha-fetoprotein level (AFP) (8). Post liver transplant, patients are subject to lifelong immunosuppression to mitigate risk of organ rejection. The aggressive recurrence of HCC has been attributed to post-transplant immunosuppression (9), though the exact mechanisms have remained unclear.

HCC is shaped by complex interactions between tumour cells and the immune system, with increasing evidence supporting the role of immune selection in driving tumour evolution. Studies on immune surveillance have shown that HCC undergoes a process of immunoediting, where immune pressure initially suppresses tumour progression but ultimately selects for resistant clones that escape immune destruction (10). Recent genomic analyses have demonstrated that HCC tumours with high immune infiltration exhibit increased selection for immune-evasive mutations, including alterations in antigen presentation pathways and immune checkpoint expression (11). Furthermore, transcriptomic studies have revealed that immune-excluded tumours—those with a non-inflamed microenvironment—tend to exhibit increased activation of oncogenic pathways that promote resistance to immune attack (12, 13) These findings highlight the dynamic nature of immune selection in HCC and its critical role in shaping tumour heterogeneity.

Post-transplant immunosuppression removes a key selective pressure exerted by the immune system, potentially enabling the outgrowth of aggressive tumour subclones that were previously constrained by immune surveillance. Prior studies have suggested that in the absence of immune control, HCC recurrence exhibits distinct molecular features, including increased genomic instability and resistance to immune checkpoint blockade (14, 15). Understanding these mechanisms is essential for refining therapeutic strategies, particularly in the selection of patients who may benefit from immune-based therapies or alternative treatment approaches.

The tumour microenvironment plays key roles in shaping tumour evolution and in determining treatment responses (14, 16). The recent clinical success of immunotherapy (13) in subpopulations of patients with previously intractable malignancies has also highlighted the importance of understanding the tumour microenvironment to identify those patients who will derive the most benefit from this class of targeted therapies (14, 16). Despite evidence of tumour genetics providing important prognostic information (13), biopsy of HCC is often omitted or inconsistently practiced in the clinical setting.

Here, we leverage genomic and epigenomic characterisation of a rare collection of baseline and recurrent tumours under conditions of immunosuppression and competence to specifically evaluate how immunosuppression alters the clonal evolution of HCC, and to document patterns of clonal evolution in this disease as a matter of general interest for the application of genomically-guided precision medicine.

## Results

### Study population

We retrieved matched formalin-fixed, paraffin-embedded (FFPE) tumour samples from 15 patients at both initial resection and at recurrence for a total of 32 samples retrieved (**Table 1**). One liver transplant recipient experienced two additional recurrences, enabling profiling of recurrent HCC lesions longitudinally. Of the 15 patients, 10 had received a liver transplant and were on immunosuppression at the time of recurrence, while the remaining 5 had been treated with liver resection alone prior to HCC recurrence. The two groups were comparable in mean age (58 years, p=0.74) and consisted entirely of male patients. The primary aetiologies of HCC included hepatitis B and C, with a higher prevalence of hepatitis B in the immune-compromised group (40%) and hepatitis C in the immune-competent group (60%). Alcohol-related HCC was observed in 10% of immune-compromised patients, whereas cryptogenic HCC accounted for 20% of cases in the immune-competent group. Preoperative tumour characteristics revealed no significant differences in alpha-fetoprotein (AFP) levels at the time of transplant or resection (p=0.84). However, the immune-competent group exhibited larger tumours compared to the immune-compromised group (5.8 ± 3.8 cm vs. 3.89 ± 3.9 cm, p=0.27). Tumour multiplicity varied, with 50% of immune-compromised patients presenting with two lesions, whereas 40% of immune-competent patients had four lesions (p=0.13). Histological grading indicated that all tumours in the immune-competent group were well-to-moderately differentiated, while only 10% of tumours in the immune-compromised group exhibited similar differentiation, with the remaining cases classified as moderately or poorly differentiated (p=0.91). Regarding vascular invasion and recurrence, microvascular invasion was present in 70% of immune-compromised and 80% of immune-competent patients (p=0.22). Major vessel invasion was observed in 10% of immune-compromised and 20% of immune-competent patients (p=0.26). Despite a longer median overall survival in the immune-compromised group (8.9 years vs. 6.4 years, p=0.27), the difference was not statistically significant. Similarly, the time to recurrence was longer in the immune-compromised group (42.03 months vs. 9.5 months, p=0.07), suggesting a trend towards delayed recurrence in this cohort. The most common sites of recurrence were the liver and lungs, with no significant intergroup differences (p=0.80). The primary treatment for recurrence varied between groups, with liver and lung resection each performed in 40% of immune-compromised patients, while immune-competent patients underwent a combination of surgical and non-surgical interventions, including radiofrequency ablation (RFA) and transarterial chemoembolization (TACE) (p=0.28). By the time of analysis, 70% of immune-compromised and 60% of immune-competent patients had died (p=0.45). These findings suggest that while tumour recurrence occurred later in the immune-compromised group, overall survival remained comparable between cohorts. We analysed the association between the clinical variables listed in **Table 1** and overall survival using univariate survival analysis with the survival package in R. However, no statistically significant associations were identified (**Supplementary Table S1**).

**Table 1:** Clinicopathological characteristics of the study participants transplanted or underwent surgical resection for HCC†.

Variable	Immune- compromised group (n=10)	Immune-competent group (n=5)	*P* value
Preoperative factors:			
Mean age (range), y	58 (49-69)	58 (48-67)	0.74
Gender, No. of men (%)	10 (100%)	5 (100%)	0.99
Aetiology			0.39
• Hepatitis B	4 (40%)	1 (20%)	
• Hepatitis C	4 (40%)	3 (60%)	
• Alcohol	1 (10%)	0 (0%)	
• MASH^†^	1 (10%)	0 (0%)	
• Cryptogenic	0 (0%)	1 (20%)	
AFP^†^, mean (range), at the time of transplant or resection (umol/L)	93.1 (4-593)	95.8 (3-309)	0.84
Tacrolimus trough level (ng/ml), median (range) between transplant and recurrence	0–3 months post LT 11.05 (3.5-15.1) 4 months post LT -recurrence) 8.55 (2-9.7)		
Tacrolimus trough level (ng/ml) mean (range), at time of recurrence	7.6 (2.9-13.2)		
Largest tumour diameter (cm), mean ± SD^†^	3.89 ± 3.9	5.8 ± 3.8	0.27
Multiplicity of lesions on the explanted liver or resected specimen, (%):			0.13
• 1 lesion	5 (50%)	2 (40%)	
• 2 lesions	5 (50%)	1 (20%)	
• 3 lesions	0 (0%)	0 (0%)	
• 4 lesions	0 (0%)	2 (40%)	
Histologic grade:			0.91
• Well-to-moderately differentiated	1 (10%)	5 (100%)	
• Moderately-differentiated	5 (50%)	0 (0%)	
• Poor-differentiated	2 (20%)	0 (0%)	
• Not available	2 (20%)	0 (0%)	
Presence of microvascular invasion on the explant or resected HCC^†^ (%)	7 (70%)	4 (80%)	0.22
Major vessel invasion	1 (10%)	1 (20%)	0.26
Vital status			0.45
• Alive	3 (30%)	2 (40%)	
• Dead	7 (70%)	3 (60%)	
Overall survival (median (range), (years)	8.9 (1.5-18.7)	6.4 (2.7-9.2)	0.27
Time to recurrence median (range), (months)	42.03 (5.9-127.9)	9.5 (6.5-17.2)	0.07
Site of recurrence:			0.80
Liver	4 (40%)	3 (60%)	
Lung	4 (40%)	2 (40%)	
Skeletal	2 (20%)	0 (0%)	
Primary treatment for recurrence:			0.28
• Liver resection	4 (40%)	2 (40%)	
• Lung resection	4 (40%)	1 (20%)	
• Radiotherapy	2 (20%)	0 (0%)	
• RFA^†^	0 (0%)	1 (20%)	
• TACE^†^	0 (0%)	1 (20%)	

† AFP, alpha fetoprotein; HCC, hepatocellular carcinoma; MASH, Metabolic-associated steatohepatitis; RFA, radiofrequency ablation; TACE, trans-arterial chemoembolization; SD, standard deviation.

### Generation of a catalogue of somatic mutations in matched pairs of HCCs

We generated somatic mutation calls using paired, matched FFPE (Formalin-Fixed, Paraffin-Embedded) samples from 15 patients at both initial tumour resection and recurrence, resulting in a total of 32 samples retrieved (**Table 1**), including two additional recurrent samples from one patient with multiple post-transplant recurrences. Whole-exome sequencing (WES) was performed at high coverage (100× for tumour, 50× for matched normal). Comparison to matched normal tissue revealed a high number of somatic mutations. FFPE samples are known to introduce artefactual mutations due to chemical modifications during fixation. These false-positive variants often follow a characteristic mutational signature (17) and typically occur at low variant allele frequencies (VAF). VAF is defined as the proportion of sequencing reads supporting a given mutation. To mitigate these errors, we incorporated FFPE-specific filtering modules into our variant calling pipeline and applied an additional VAF threshold to exclude variants likely to be artefactual. Given that FFPE-induced mutations exhibit characteristic patterns of DNA damage and repair, this dual-filtering strategy effectively reduced their prevalence in the final mutation set (**Supplementary Figure S1A**). As expected, the filtering also decreased the overall number of detected mutations, resulting in variable retention rates of initially called variants following VAF-based exclusion (**Supplementary Figures S1B, C**).

**Figure 1:**
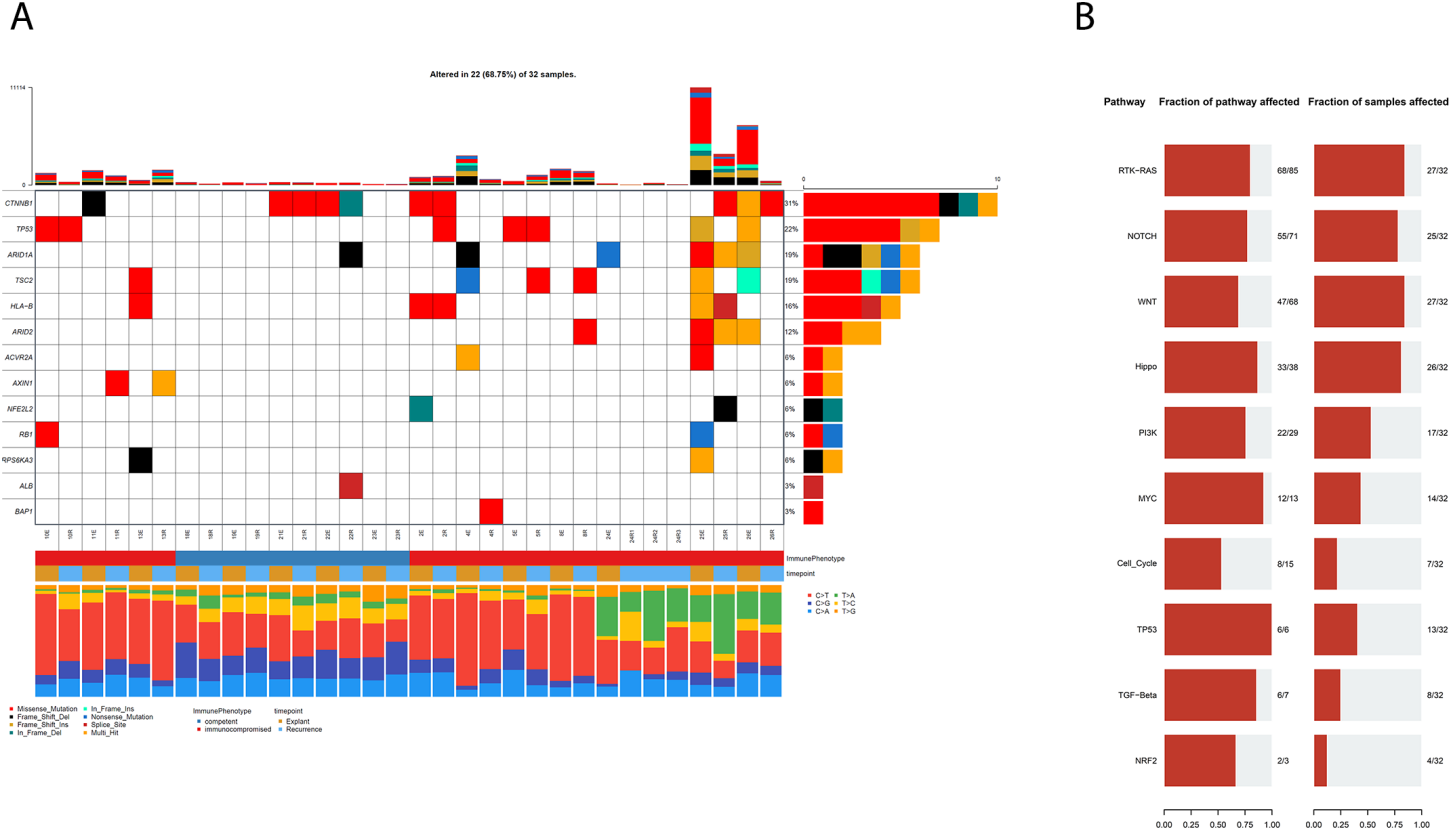
**(A)** Co-mutation Plot showing mutations in known HCC driver genes in our cohort of FFPE HCCs following FFPE specific filtering and the application of a secondary variant allele frequency filter. **(B)** Representations of pathways associated with observed mutations, in terms of the number of genes in a pathway that are mutated and the fraction of samples with mutations in the relevant pathways.

Because multiple samples were derived from some patients, the dataset lacked full independence, limiting our ability to apply *de novo* driver discovery tools like dNdScv (18). We therefore focussed our analysis on previously characterised HCC and pan-cancer driver genes (18). Mutations in known HCC drivers were identified in 68% (22/32) of samples, though they occurred less frequently in tumours from immunocompetent patients (**Figure 1A**).

All samples had mutations in genes previously implicated in pan-cancer oncogenesis, suggesting that these variants may represent alternative routes to tumour development in some patients (mutation data in **Supplementary Tables S2**).These findings suggest that genomic assays including known pan-cancer driver genes may be clinically useful in the management of HCC using precision medicine. We identified RAS-RTK as the most frequently mutated pathway, both in terms of percentage of genes mutated within each canonical pathway as well as in the proportion of affected samples (**Figure 1B**). The other most mutated pathways included the NOTCH, Wnt and HIPPO signalling pathways, in concordance with established knowledge of HCC (19).

### Extensive genomic divergence defines HCC evolution

In order to characterise the clonal structure and variation of mutations in our HCC cohort, we integrated the variant allele frequency and high confidence mutation calls with allele-specific absolute copy number calls generated using Sequenza. This was then used in conjunction with PyClone_VI (20), a variational bayes inferential method of the original PyClone algorithm (21), to estimate the cancer cell fraction (CCF) of each mutations and to then cluster mutations into distinct clones based on their CCF distribution.

**Figure 2:**
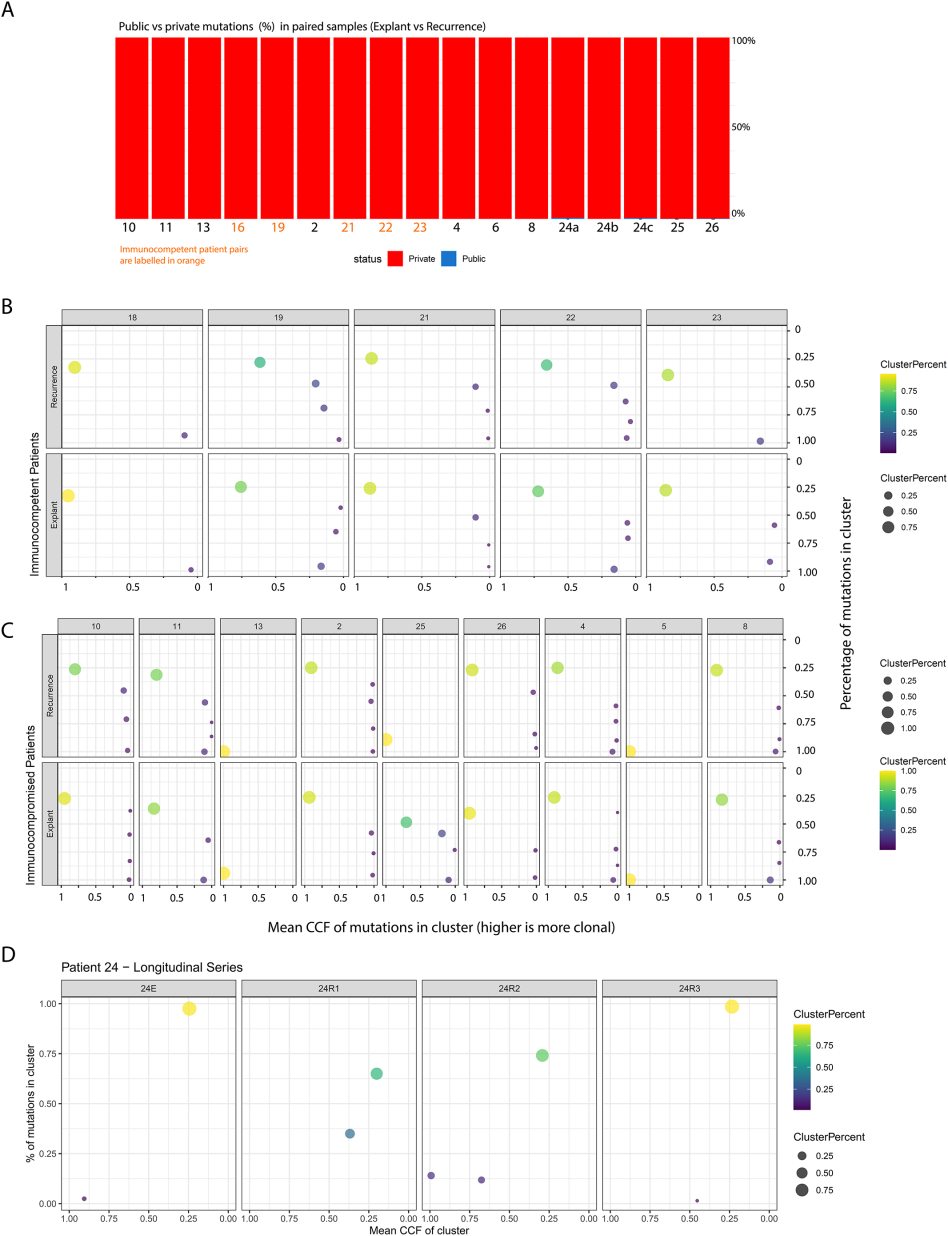
**(A)** Breakdown of mutations in each patient, classified as *private* (found only in either the primary or recurrent tumour) or *public* (shared between both). The X-axis shows patient identifiers, and the Y-axis shows the percentage of all mutations found in each patient at either timepoint. **(B, C)** represent clone-level views of the primary and recurrent tumours for each patient, with the Y-axis indicating the percentage of all tumour mutations belonging to clones at a given Cancer Cell Fraction (X-axis). Point size and colour reflect the relative size of each clone based on mutation percentage. These results are split into panels showing: (1) immunocompetent control tumours across timepoints, (2) immunocompromised tumours across timepoints, and finally **(D)** longitudinal samples spanning multiple years from one immunocompromised transplant recipient.

Initial comparisons between baseline and recurrence samples revealed that the vast majority of mutations were private (i.e., found only at one timepoint, **Figure 2A**), indicating marked evolutionary divergence. This prompted us to assess whether the low mutational overlap observed between baseline and recurrent samples might be an artefact introduced by our stringent VAF-based filtering of formalin-fixation-related errors. We reasoned that if the observed lack of shared mutations were primarily due to filtering, then the overlap between matched baseline-recurrence pairs should be no greater than that seen between tumours from unrelated individuals. To test this, we analysed whole-exome sequencing data from 360 HCC cases in TCGA and calculated the distribution of mutational overlap across all possible pairwise combinations (n = 129,600).

The percentage of overlap between our matched patient samples was significantly higher in comparison to the distribution seen in the unmatched control samples, suggesting that our filtration strategy did not erroneously inflate the extent of evolutionary divergence we observed (**Supplementary Figure S1D**, p < 2.2e-16, Wilcoxon’s Rank Sum Test).

We subsequently characterised the clonal composition of our tumours, by clustering mutations into distinct clones based on their Cancer Cell Fractions (CCF) using a variational Bayesian inference version of the widely used PyClone algorithm. This approach is based on the principle that due to the nature of chromosomal segregation during mitosis and inheritance of pre-existing and novel variants by daughter cells, mutations that occur at similar allele frequencies are likely to co-exist in the same cellular populations. Without multi-region data, however, we decided not to perform phylogenetic reconstruction of clonal relationships within our samples, since mutation ordering is challenging in the absence of multi-region data. These findings are consistent with polyclonal seeding of recurrences in both immunocompetent and immunocompromised patients, a pattern previously described in prostate cancer metastases previously (22). The clonal structure in these tumours specifically demonstrated the presence of small clonal populations at high CCF and the large majority of mutations in a sample being present subclonally (**Figure 2B**). Further, in the one patient from whom we profiled multiple post-transplant recurrent samples across a timespan of years, we observed marked temporal shifts in clonal structure (**Figure 2C**). Taken together, the level of heterogeneity and evolution observed in these tumours suggests that repeat biopsies—ideally incorporating multi-region or spatially resolved sampling—may be necessary at recurrence to guide the selection of effective targeted therapies.

**Figure 3:**
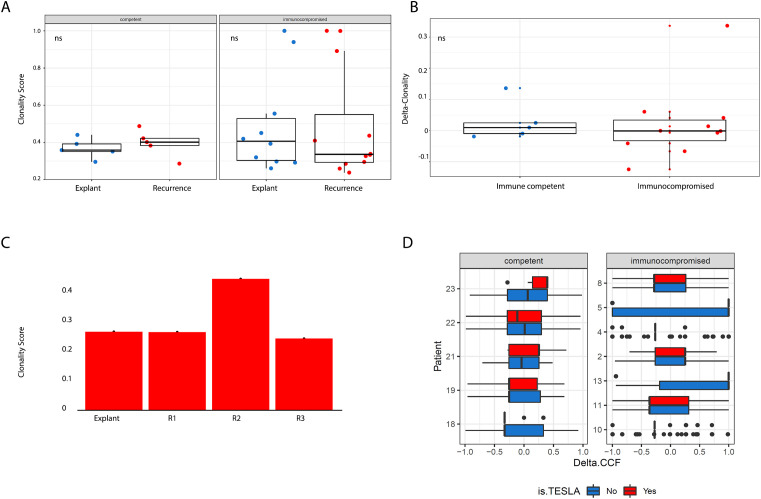
**(A)** Boxplots showing breakdowns of clonality scores (Y axis) across patient group and timepoint (X axis). More clonal tumours have higher clonality scores. We did not observe significant differences across timepoints when comparing by patient group or across patient group when comparing by timepoints. **(B)** Boxplots showing the magnitude of change in clonality scores (Y axis) between timepoints across patient group (X axis). We did not observe significant differences across timepoints when comparing by patient group or across patient group when comparing by timepoints. All tests pertaining to this figure were conducted using a two-sided Wilcoxon’s Rank Sum Test **(C)** Longitudinal series of samples profiled from a patient at multiple post-recurrence timepoints, no statistically significant variation in clonality score was observed. **(D)** Distribution of delta CCF for all mutations in a pair of patient samples, which revealed no significant distributional shifts. Boxplots illustrate patterns of shifts in allele frequency for all mutations present in either the primary or recurrent sample for each patient, segregated by neoantigen status (predicted neoantigens are in red, predicted non-neoantigens are in blue).

To quantitatively assess clonal architecture, we calculated a clonality score for each sample. This score was defined as the sum of each mutation’s cancer cell fraction (CCF) weighted by its frequency, generating a value between 0 (entirely subclonal) and 1 (entirely clonal). Notably, we observed no statistically significant differences in clonality between immunocompetent and immunocompromised patients, either at baseline or at recurrence, nor in the extent of change between these timepoints (**Figures 3A, B**). This remained true even in the longitudinal series from a single patient (**Figure 3C**).

Taken together, these findings indicate that immunosuppression did not influence the evolutionary trajectory of these tumours, suggesting that immunoediting was not an active process during transplantation and subsequent recurrence. We initially hypothesised that if these tumours had not already evolved mechanisms to evade immune selection before the onset of immunosuppression, we would observe either an expansion of pre-existing neoantigens (novel proteins arising from somatic mutations that the adaptive immune system may recognise as non-self due to significant divergence from their wild-type counterparts) or a disproportionate enrichment of neoantigens in the mutational repertoire of recurrent tumours. However, the absence of these patterns suggests that immune escape had already occurred prior to transplant-associated immunosuppression.

Immunoediting entails elimination of cancer cells carrying immunogenic mutations, followed by selection and expansion of cancer cells capable of evading the immune system through a wide range of permanent, genetic, lesions or other transient mechanisms such as the upregulation of immunosuppressive checkpoint proteins (23, 24). Immunotherapy has emerged as a promising strategy through the blockade of immune checkpoints, such as CTLA-4 and PD-1, which can rebalance the Immunoediting due to immunogenic mutations in favour of tumour elimination (25).

Neoantigen calling is not an exact science, and the best state-of-the-art neoantigen calling criteria only manage to achieve about 70% sensitivity at 98% specificity based on integration of predicted affinity of binding to Class I MHC alleles and sequence similarity to pathogenic peptides and dissimilarity to the self-peptidome (26). As a result, our findings are likely to miss real changes at the expense of avoiding false positive findings.

Using the Antigen Garnish pipeline, we generated neoantigen calls and then further applied the affinity criteria developed by the TESLA consortium (the current state-of-the-art, as described earlier) to yield a stringently filtered set of candidate neoantigens for the vast majority of samples. For a subset of samples (n = 5), our HLA caller reported insufficient numbers of paired reads to call HLA type. For secondary analyses, we used more traditionally employed thresholds for binding affinity; 100nM (strong binders) and 500nm (weak binders) or less. We then quantified, for each mutation, the delta-CCF between recurrent and baseline samples and compared their distributions between candidate neoantigens and control mutations. In accordance with our general, mutanome-wide finding that there was no influence of transplant-related immunosuppression on the evolution of recurrent HCCs, we found no evidence for selective expansion of neoantigens in immunocompromised samples compared to controls (**Figure 3D**).

### The acquisition of genetic lesions associated with immune evasion explains the lack of effect of immunosuppression on neoantigen evolution

However, these findings were consistent with multiple alternative explanations; first, any immune response that was narrowly targeted against only a few neoantigens would not affect the overall distribution, especially when the total number of candidate mutations that may produce neoantigens is high. Due to a lack of TCR sequence profiling, we were unable to estimate the breadth of the T-cell immune response and could not empirically assess whether this was the case. Secondly, another possibility is that neoantigens may be regulated epigenetically or transcriptionally. While our cohort lacked transcriptomic data, the availability of matched Illumina EPIC array profiles for most of our samples enabled us to test for differences in promoter methylation at neoantigens and non-neoantigens alike. Again, we failed to see significant differences upon lifting the immune selection pressures in the immunosuppressed patients (**Supplementary Figure S1C**). However, the lack of transcriptomic data prevents us from accounting for changes in the transcriptional states of neoantigens that may be regulated by mechanisms other than DNA methylation, or changes in non-genomic mechanisms that regulate anti-tumour immunity.

The remaining explanation was that the acquisition of genetic alterations associated with immune evasion had created a state of enduring immune escape in these tumours before transplantation. To test this hypothesis, we established a gold-standard catalogue of experimentally validated hits from CRISPR screens that used indel-producing sgRNAs to identify genes that, when disrupted, resulted in direct resistance to CD8 T-cell mediated killing (27), as well as genes known to be canonically involved in antigen processing and presentation.

Since the functional interpretation of missense mutations can be challenging, we reduced our analysis to mutation types typically associated with loss of function (nonsense mutations, frameshifts, other indels). We observed that 19/22 tumours in the transplant group contained at least one loss of function mutation in genes known to confer resistance to T-cell mediated destruction, as opposed to only 2/10 of the immunocompetent tumours.

Amongst the groups of genes impacted recurrently were multiple components of antigen presentation machinery (the class 2 MHC genes *HLA-DRA, HLA-DQA, HLA-DQB*, as well as the main transcriptional activator of MHC-II genes (CIITA), and the essential invariant MHC-I component B2Mand the primary antigen transport protein, TAP1, which is essential for the formation of functional MHC-I complexes), a significantly large repertoire of core interferon transcription factors) (IRF2/IRF8) and key signal transduction kinases amongst others (JAK2 and PRKCD, both of which are involved in post-translational modifications of STAT1, a master regulator of transcriptional activation of genes transcribed as part of the interferon response (28).

**Figure 4:**
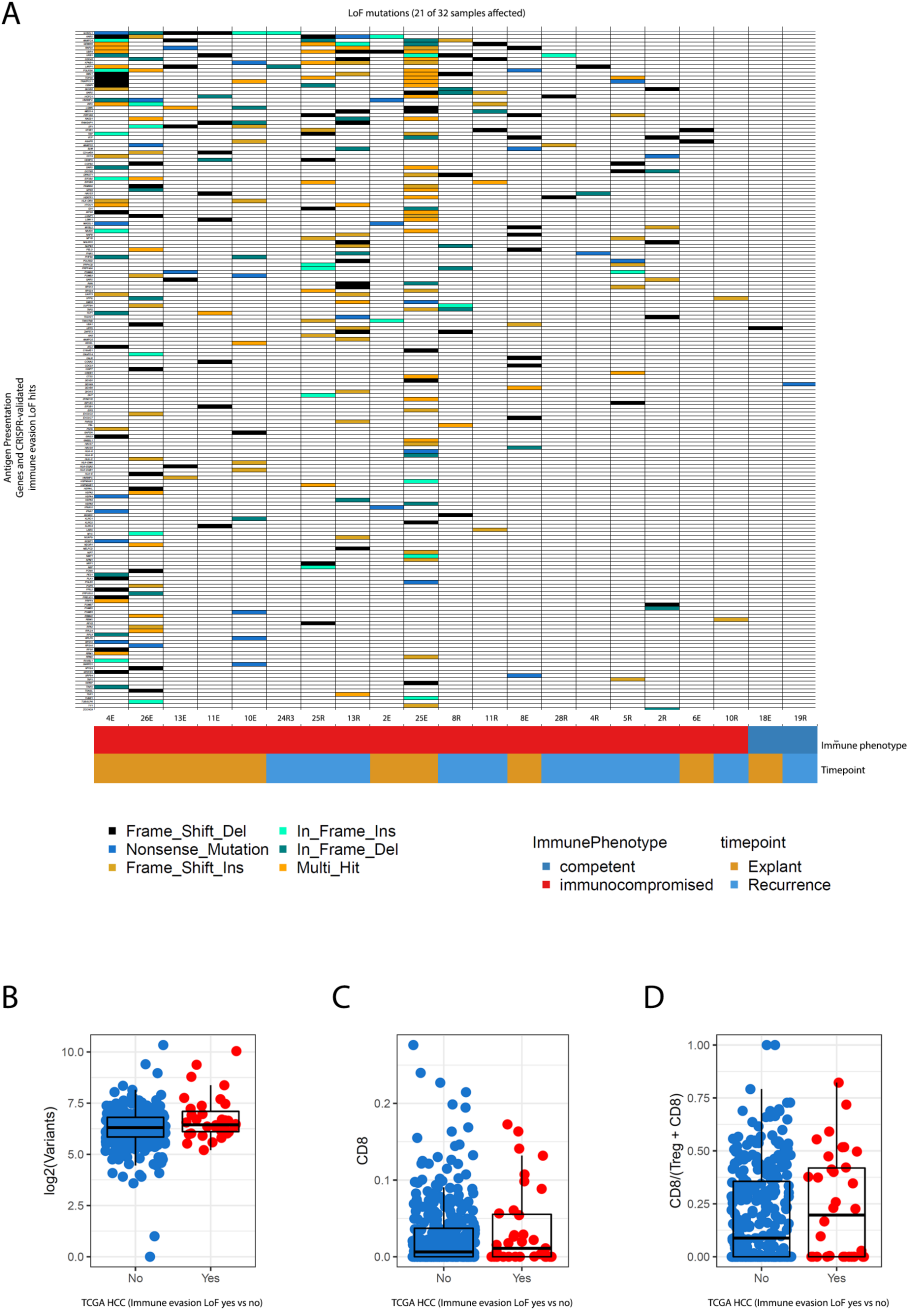
**(A)** Co-mutation plot shows patterns of loss-of-function mutations in genes experimentally validated to confer a resistance to CD8 T-cell mediated killing upon inactivation in CRISPR screens and canonically recognised antigen presentation pathway genes. These mutations are frequent and numerous in patients that went on to receive systemic immunosuppression, both at baseline, and upon recurrence. **(B–D)** represent relationships between parameters of immunogenicity (mutational burden, CD8 infiltration, CD8/Treg ratios) in TCGA HCC data for the same catalogue of genes. Tumours are segregated by whether they possess immune-evasion conferring LoF mutations or not. Statistical estimates are from Wilcoxon’s Rank Sum Test.

This offered a potential explanation for why immunosuppression did not affect the behaviour of neoantigens; quite simply, prior genetic histories had already nullified the effect of any future modulation of immune selection pressures (**Figure 4A**).

While our cohorts were limited by sample size, we reasoned that if our observation of enrichment for immune-escape genomic alterations pre-treatment was a generalisable finding, large cohorts of excised HCC would also demonstrate the same pattern. Therefore we also investigated the extent to which these mutations were present in resected primary HCC samples from the TCGA to evaluate whether genetic mechanisms of immune escape are commonly present at baseline. Using the same stringent criteria as described above, we found that nearly 10% of TCGA HCCs possess loss-of-function (LoF) mutations in at least one of the immune evasion genes surveyed, which is no doubt a conservative estimate due to our stringency. The inclusion of missense mutations in these genes further expanded this number to 55% of TCGA HCCs as a liberal upper bound.

Traditionally, selection for immune escape-related events has been associated with the presence of an inflamed antitumour immune microenvironment, often in the presence of a high burden of neoantigens (29). Indeed, we observed that most of the samples in our transplant cohort had much higher mutational burden (**Figure 1A**) even at baseline before exposure to immunosuppression. This higher mutational burden tracked with the presence of immune evasion LoF alterations, and was also present in the matched recurrent HCCs post-transplant.

In TCGA HCCs however, the presence of immune evasion LoF alterations was not associated with increasing mutational burden but remained strongly associated with increased CD8 infiltration as assessed using DNA methylation through MethylCIBERSORT, and higher CD8/Treg ratios (**Figures 4B–D**, p < 0.05 for the two latter metrics Wilcoxon’s Rank Sum test).

## Conclusions

The temporal profiling of tumours under conditions of different activity in the immune microenvironment can reveal how tumour evolution is shaped by the immune system. In this study, we leveraged the availability of patients who had tumours recur under conditions of immunosuppression to observe these trends, as well as gain an understanding of evolutionary divergence in HCC tumours in general from the perspective of clinical management.

Our observation of extensive evolutionary divergence between baseline and recurrent tumours indicates the need for re-biopsy upon recurrence to ensure that the treatments selected are targeting lesions found within the tumour. Where recurrent or worsening disease is marked by multiple metastases, approaches that are based on whole-exome-sequencing of the plasma, or other approaches such as CAPP-seq with panels designed to target druggable genes, may be a useful substitute for biopsies that are hard to obtain.

The complex clonal structures of these tumours, as well as the strong propensity for subclonal mutagenesis, both point to intratumour heterogeneity being a strong factor in the clinical behaviour of these tumours. This is consistent with multi-region sequencing studies of HCC at a single time point that reveal a similar degree of heterogeneity (14, 16, 30–36).

Finally, the high burden of immune evasion mutations in neoantigen-rich tumours implicates a genomic basis for a state of immune escape that may result in the failure of immune checkpoint blockade. We therefore suggest that the management of HCC regularly involve sequencing to detect the presence of these alterations in order to carefully select patients that may respond to immune checkpoint blockade. Multiple clinical trials of ICB monotherapies in HCC have indicated overall response rates between 20-32% (37, 38) in patients. The role of checkpoint inhibitors in the treatment of hepatocellular carcinoma is evolving and expanding (39, 40). An essential step in expanding the use of CPI in unresectable HCC and recurrence is recognising the abundance of pre-existing immune evasion mutations in tumours. This in turn may facilitate more efficient use of these therapies.

Our findings align with studies in other cancers demonstrating that immune selection pressures drive tumour evolution and resistance to immunotherapy (25). Similar patterns of immune escape have been observed in metastatic melanoma and non-small cell lung cancer, where tumours acquire genetic alterations that impair antigen presentation and T-cell recognition (24, 29, 36). Comparing HCC with these malignancies highlights the broader implications of immune-driven tumour evolution and reinforces the need for multi-modal treatment approaches (41, 42).

Our results suggest that tumour-intrinsic factors, rather than host immunity, may be the dominant force driving HCC recurrence, explaining why immunosuppression failed to alter tumour evolution. One potential mechanism is inherent resistance to immune editing, where tumours had already undergone immunoediting prior to transplantation, selecting for clones that could evade immune-mediated destruction (13, 43). Previous studies have shown that tumours frequently develop genetic and epigenetic mechanisms that disable antigen presentation and modulate immune checkpoint expression, rendering them resistant to immune attack irrespective of immunosuppressive status (15). Given that immune checkpoint inhibitors (ICIs) have shown efficacy in a subset of HCC patients with intact antigen presentation pathways (44), our findings suggest that only select patients may benefit from these therapies. Future research should explore combination approaches, integrating ICIs with epigenetic modulators or kinase inhibitors to enhance anti-tumour immunity, particularly in immune-excluded tumours (45). Furthermore, the role of HCV-driven oncogenesis in shaping immune evasion landscapes remains an important avenue for investigation, as regional disparities in HCV prevalence and antiviral access could impact both HCC evolution and treatment strategies (46, 47). A deeper understanding of these molecular interactions could inform tailored therapeutic interventions and precision oncology approaches for HCC patients across different disease contexts.

Our contextualisation of how immune escape works in HCC is a useful contribution to this end, especially when these patterns are reflected in much larger cohorts of resected HCC. Our pilot study points to the importance of extensive spatial and temporal genomic characterisation of HCC to guide personalised therapy.

### Limitations

While our study provides valuable insights into HCC evolution under immune selection pressures, it is limited by sample size and the absence of transcriptomic data. Future studies should integrate RNA sequencing and spatial transcriptomics to assess how immune-modulatory changes at the transcriptional level influence tumour progression (31).

## Methods

### Patient population

Patients who underwent liver transplant or surgical resection for hepatitis B/C or alcohol-induced HCC between 2002 and 2016 were included in our study. Ten patients who underwent liver transplant (n=10) or surgical resection (n=5) were included in our study. Patients with a maximum of 4 viable lesions of the primary tumour were included. For both groups, archived formalin-fixed, paraffin-embedded (FFPE) samples available were retrieved. In case of more than one HCC lesion, only the largest dominant lesion was retrieved to obtain DNA. DNA was obtained from the FFPE samples using the QIAamp DNA FFPE Tissue Kit (QIAGEN) following manufacturer’s instructions. Characteristics of the tumours on the explant or resected lesions, including grade, size, number of tumours, the presence of microvascular invasion and associated AFP at the time of transplant or resection were documented. For the recurrent lesions, the date of recurrence, the number of lesions identified, the AFP peak and the type of therapy used to treat the recurrent lesions were also retrieved. For the transplant cohort, the trough level of the immunosuppressant medication from the time of transplant to the time of recurrence was also documented. Patient clinicopathological characteristics are summarised in **Table 1**.

### Statistical analysis

Comparative statistical analyses of clinical characteristics between the immune-compromised and immune-competent groups were performed using non-parametric tests. Continuous variables were summarised as means with standard deviations or medians with interquartile ranges, and group differences were assessed using the Wilcoxon rank-sum test. Categorical variables were compared using Fisher’s exact test due to the small sample size. The association between clinical variables and overall survival was explored using univariate Cox proportional hazards models with the coxph() function in the survival package (https://cran.r-project.org/package=survival (48)in R (v3.8-3).

Details of the statistical methods are outlined in the Methods section, described in-line in the text, and summarised in the figure legends. Proportions were analysed using Fisher’s exact test, and comparisons of distributions were performed using the Wilcoxon rank-sum test.

Briefly, differences in clonality scores were evaluated using two-sided Wilcoxon rank-sum tests. The extent of mutational overlap between paired baseline-recurrent samples versus unrelated TCGA controls was assessed using Wilcoxon tests. Associations between immune escape mutations and markers of immune infiltration (e.g., CD8+ T-cell levels, CD8/Treg ratios) in the TCGA dataset were also evaluated using Wilcoxon rank-sum test.

### Ethics statement

This study was conducted in accordance with the Declaration of Helsinki and approved by the University Health Network (UHN) Research Ethics Board (REB#22-5006). Given the retrospective nature of this study, a waiver of consent was granted for the collection of retrospective data and tissue samples. While retrospective studies offer valuable insights, they are inherently subject to potential selection biases, including the availability of archived specimens and variations in clinical follow-up. To mitigate these biases, we implemented standardised inclusion criteria and validated our findings against publicly available datasets where applicable.

### Whole exome sequencing and variant calling

*Methylation profiling:* DNA was extracted from FFPE samples using QIAamp DNA FFPE Tissue Kit (Qiagen) following manufacture’s protocol and quantified using Qubit 4 fluorometer (ThermoFisher). DNA (500ng) was bisulfite-converted and processed on Infinium Human Methylation EPIC BeadChips (Illumina Inc.) (41). 

*Whole exome sequencing:* Exome capture was performed using Agilent exome capture kit on extracted DNA (10 ng). Libraries were prepared using Agilent SureSelect XT-HS kit and hybridised to Agilent human all exon v7 panel. Sequencing included 125 cycle paired-end reads from Illumina HiSeq 2500 at 100X for tumour samples and 50X for normal tissue, followed by exome alignment and mutation calling (49).

Fastq files were aligned to hg19 using BWA-mem (50) and were processed using Samtools (51) to remove duplicate reads for all samples. For each sample, we sequenced the explant/baseline sample, the recurrent sample, and matched normal tissue, sourced from adjacent pathologically normal liver tissue at the time of explant resection. Somatic mutations (both SNVs and short Indels) were then called using MuTect2 (52).

To mitigate artefactual mutations in FFPE samples, the FFPE module in MuTect2 was applied, followed by additional filtering to remove mutations with a variant allele frequency below 10% based on MuTect2’s empirical VAF output.

The neoantigen pipeline used, Antigen Garnish (53), required hg38 input to accurately call neoantigens. We therefore used Picard (http://broadinstitute.github.io/picard/) to lift over variant calls to hg38 using the UCSC chain file. Our hg19 callset was annotated using Annovar (53) to annotate these mutations for downstream analyses and functional annotation using the maftools R package (54), and we used snpEff 4.5 (55) to annotate VCFs for neoantigen calling from our lifted over callsets. Mutational signature analysis was performed using DeconstructSigs (53) using the updated COSMIC mutational signatures database for reference (54).

### Copy number calling and clonality analyses

We used Sequenza-utils to create input files for Sequenza (56) from tumour and normal BAM files, followed by segmentation and probabilistic modelling to estimate purity, ploidy, and absolute copy number combinations that best fit the observed data. The absolute copy numbers from the top-scoring solution, along with variant allele frequencies estimated during SNV calling, were used to cluster variants into clones using PyClone-VI, specifying an upper limit of 15 subclones and 15,000 iterations.

### Neoantigen calling and estimation

Class I HLA types were called from the normal tissue samples for each patient using OptiType (57) at four-digit resolution.

The Antigen Garnish (53) package was used to estimate the Class I MHC binding potential of nonameric peptide (composed of nine amino acids) sequences containing somatic mutations, based on hg38-lifted and annotated VCF files generated in previous steps. Peptides meeting the stringent affinity criteria established by the TESLA consortium, a gold standard for immunogenic neoantigens, were selected. The distribution and clonal dynamics of predicted neoantigens were then compared to non-neoantigens to assess the impact of immunosuppression on neoantigen evolution.

### Analysis of DNA methylation profiles at neoantigens

We generated Illumina EPIC array data for our samples that were processed uniformly using the minfi R package via the ssNoob normalisation pipeline (58). We then summarised the methylation at upstream regulatory region probes (formally designated as TSS200, TSS1500, and 1^st^ Exon according to Illumina convention) using the average and the median, given the general association of these probes with repression of gene expression. We then examined methylation shifts at predicted neoantigens and non-neoantigens as a function of timepoint and immunosuppression status.

### Analysis of immune evasion genes

We defined a catalogue of immune evasion genes by combining gene sets of antigen presentation genes from the Kyoto Encyclopaedia of Genes and Genomes KEGG) (59), and genes with loss-of-function hits implicated in resistance to CD8 T-cell mediated destruction in CRISPR screens Patel et al (27). To define loss of function mutations in our whole exome dataset, we only included variants classified as nonsense, frameshifts, other indels and nonstop mutations. Proportions were compared using a Fisher’s Exact Test. TCGA mutation calls were obtained from the MC3 callset via SAGE Synapse and were restricted to the same categories of variants. Associations of LoF mutations in immune evasion genes with immune infiltration (CD8 levels and CD8/Treg ratios) and mutational burdens were tested using Wilcoxon’s Rank Sum Tests.

## Data Availability

Methylation and Whole Exome Sequencing data supporting this study’s findings are available upon reasonable request from the corresponding author, in compliance with institutional ethics regulations. External researchers interested in accessing the data should submit a formal request via email to the corresponding author, including a brief description of the intended research use, institutional ethics approval (if applicable), and a signed data-sharing agreement. Upon approval, the data will be made available through a secure repository under controlled access to ensure compliance with patient confidentiality and ethical guidelines.

## Supplementary material

Supplementary Figure 1
**(A)** Analysis of artefactual mutational signatures and the percent of mutations mapping to them with caller-specific FFPE filtering, and caller specific FFPE filtering combined with an additional VAF filter (removal of mutations at less than 10% VAF), respectively. **(B, C)** are bar graphs that show the total numbers of mutations and percentages before and after the additional VAF filter was applied to our call set. **(D)** is a boxplot showing the percentage of overlap in mutanomes (Y axis) in our matched patient cohort when compared to a distribution of overlaps in mutanomes between unmatched patient samples derived from different individuals in the TCGA HCC cohort (X axis). P < 2.2e-16, Wilcoxon’s Rank Sum Test. **(E)** Boxplots show distributions of mean CpG methylation beta values at upstream regulatory regions (Y axis) of genes predicted to encode neoantigens (red) or not (blue) by timepoint (primary tumour/recurrence) (X axis), each panel represents one patient, with the immunocompetent/compromised status of the patients labelled.
