# Supplementary figures and images for: Evolutionary dynamics of recurrent hepatocellular carcinoma under divergent immune selection pressures
Source: Front Oncol. 2025 Aug 4;15:1537087. doi: 10.3389/fonc.2025.1537087 (PMC12359061; doi:10.3389/fonc.2025.1537087)

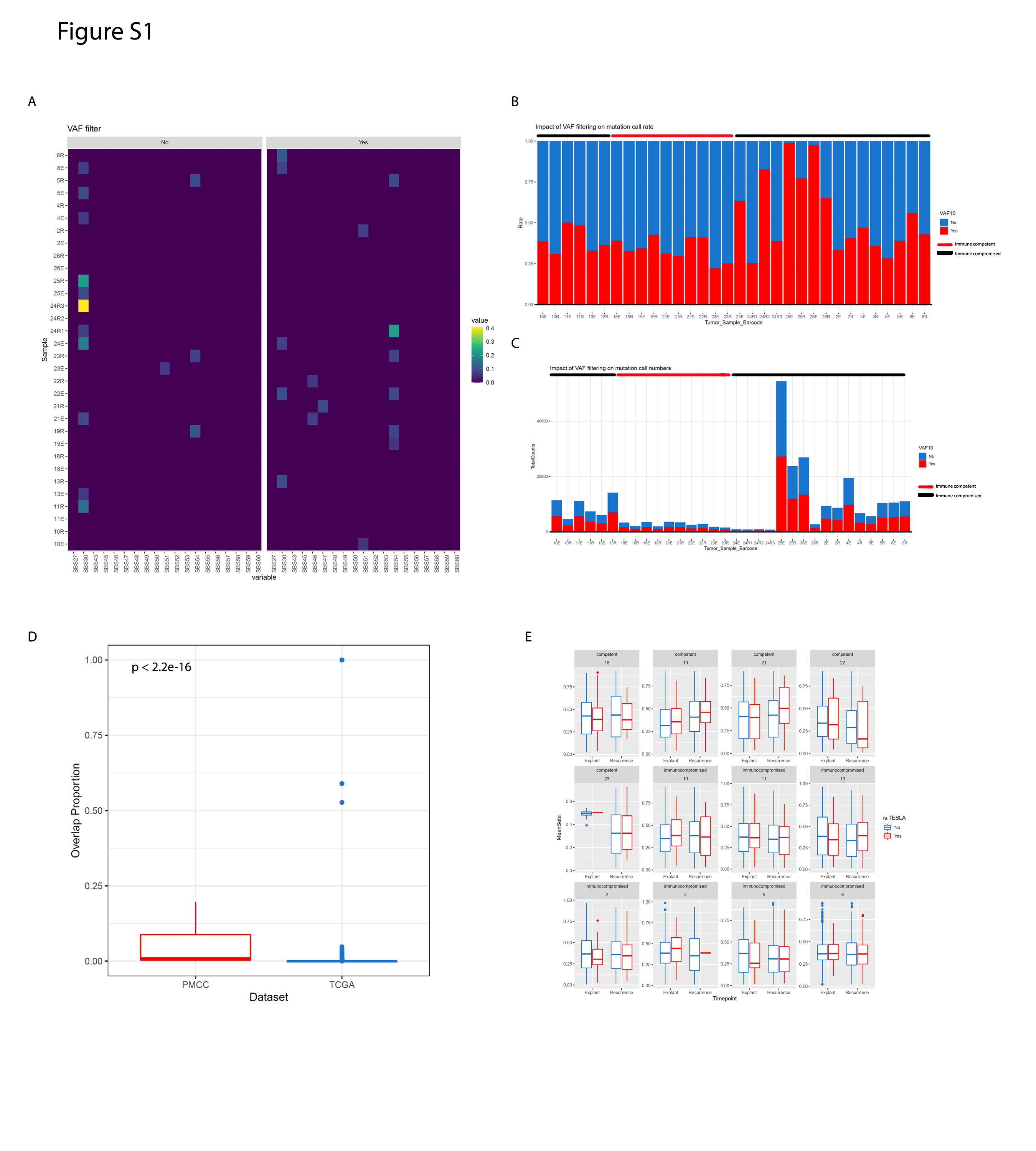

Supplement: Supplementary Figure 1 — (A) Analysis of artefactual mutational signatures and the percent of mutations mapping to them with caller-specific FFPE filtering, and caller specific FFPE filtering combined with an additional VAF filter (removal of mutations at less than 10% VAF), respectively. (B, C) are bar graphs that show the total numbers of mutations and percentages before and after the additional VAF filter was applied to our call set. (D) is a boxplot showing the percentage of overlap in mutanomes (Y axis) in our matched patient cohort when compared to a distribution of overlaps in mutanomes between unmatched patient samples derived from different individuals in the TCGA HCC cohort (X axis). P < 2.2e-16, Wilcoxon’s Rank Sum Test. (E) Boxplots show distributions of mean CpG methylation beta values at upstream regulatory regions (Y axis) of genes predicted to encode neoantigens (red) or not (blue) by timepoint (primary tumour/recurrence) (X axis), each panel represents one patient, with the immunocompetent/compromised status of the patients labelled. [file Image1.tif]
